# Navigating the Myocarditis Challenge: Advanced Approaches for PD-1 Inhibitor Trials

**DOI:** 10.7759/cureus.79195

**Published:** 2025-02-17

**Authors:** Oghenetanure R Enaworu

**Affiliations:** 1 Cancer Research Center, University of California Irvine, Irvine, USA

**Keywords:** cancer immunotherapy, clinical trial, immune-related myocarditis, oncology, pd-1 inhibitors

## Abstract

The introduction of programmed cell death protein 1 (PD-1) inhibitors has revolutionized cancer immunotherapy, offering new treatment options for patients with refractory malignancies. However, immune-related myocarditis (IRM), occurring in approximately 1.14% of patients receiving immune checkpoint inhibitors (ICIs), presents a significant challenge, with mortality rates of 25-50%, necessitating early detection and effective management.

Glucocorticoids, the standard treatment for IRM, complicate management by suppressing T-cell activity and diminishing PD-1 efficacy. Diagnosing IRM remains difficult, as endomyocardial biopsy, the gold standard, is often impractical, while cardiac MRI, commonly used as an alternative, has limitations (sensitivity 0.66, specificity 0.73).

To improve IRM management, integrating routine cardiac MRI with biomarker assessments, supported by AI-driven diagnostic tools, can enhance early detection and reduce diagnostic uncertainty. Flexible clinical trial protocols should allow PD-1 therapy resumption post-IRM, independent of glucocorticoid duration. Additionally, creating separate trial arms for patients recovering from long-term glucocorticoid use can help isolate its impact on treatment response, while advanced statistical models can account for glucocorticoid duration, ensuring robust efficacy assessments.

Finally, educating trial stakeholders on these strategies is essential for optimizing patient safety and generating reliable clinical outcomes in PD-1 inhibitor trials. Implementing these approaches will enhance the management of IRM while preserving the therapeutic benefits of PD-1 inhibitors.

## Editorial

The introduction of programmed cell death protein 1 (PD-1) inhibitors in cancer immunotherapy has marked a major milestone, offering new hope for cancer patients who have progressed despite traditional therapies. PD-1 inhibitors, a subclass of immune checkpoint inhibitors, block the PD-1/PD-L1 interaction to restore T-cell activity and enable the immune system to target and destroy cancer cells.

While immune-related myocarditis (IRM) has a reported prevalence of 1.14% in a multicenter study of patients treated with immune checkpoint inhibitors (ICIs), including PD-1 inhibitors [1], this rate can vary depending on the patient population, therapy type, and specific trial settings. Despite this variability, IRM can rapidly progress to fatal outcomes, with mortality rates of 25-50% [2], making early detection critical. Non-specific symptoms such as fatigue, chest discomfort, and palpitations, along with biomarkers like troponin, can lead to unnecessary trial adjustments, such as prematurely withholding PD-1 inhibitors due to patient safety concerns. Alternatively, delays in diagnosis can result in life-threatening adverse events like arrhythmias and heart failure, compromising patient safety. This creates a dilemma for principal investigators, who must balance the risk of severe complications with the potential harm of prematurely stopping PD-1 inhibitors, a decision that depends on clinical judgment.

In addition, the treatment of IRM typically involves glucocorticoids, which interfere with the efficacy of PD-1 inhibitors by inhibiting inflammatory cytokines and suppressing T-cell activity, thus requiring the withholding of PD-1 inhibitor dosing during the trial. Glucocorticoid use in PD-1 inhibitor trials follows trial protocols with predefined dose thresholds that require PD-1 therapy to be paused during moderate to severe IRM symptoms and resumed once glucocorticoids are tapered to physiological doses and symptoms resolve completely or to mild or baseline levels. However, prolonged glucocorticoid use may prevent the resumption of PD-1 inhibitor therapy, creating a dilemma despite the patient's initial eligibility and limited alternative treatments after progression. This creates tension between patient safety, protocol adherence, and addressing the needs of cancer patients seeking better outcomes with PD-1 inhibitor trials after progression on standard care.

Differentiating myocarditis from other non-specific cardiac conditions is challenging in cancer patients with various risk factors. While endomyocardial biopsy is the gold standard for diagnosis [3], it is often not feasible in clinical trials due to safety concerns related to the procedure (e.g., bleeding, infection), invasiveness, and logistical issues. Cardiac MRI offers a non-invasive alternative to endomyocardial biopsy (EMB) for diagnosing IRM, with a sensitivity of 0.66 and specificity of 0.73 [4]. However, its accuracy decreases in patients with arrhythmias or when imaging is delayed. Moreover, MRI is not always performed routinely in trials due to resource limitations, logistical constraints, and trial protocol design, with intervals sometimes extending to 8 or even 12 weeks, complicating timely diagnosis and intervention.

To address diagnostic challenges and glucocorticoid overuse in PD-1 inhibitor trials, enhanced protocols, such as cardiac MRI scans and troponin biomarkers when symptoms arise, can enable timely and accurate myocarditis detection. Additionally, cardiac magnetic resonance (CMR) imaging requires expertise to minimize bias; thus, the use of artificial intelligence (AI) techniques, including supervised, unsupervised, and reinforcement learning, is crucial for improving accuracy and reducing bias [3]. A report indicates that glucocorticoid treatment, when appropriately administered, does not compromise the efficacy of PD-1 inhibitors [5]. Consequently, establishing clear decision algorithms for glucocorticoid use in PD-1 therapy trials is vital, given the tension between patient safety concerns and the limited treatment options available. To tackle the challenge of premature removal from PD-1 therapy trials due to glucocorticoid use, flexible trial protocols should be implemented, allowing patients to resume therapy once side effects are managed, regardless of glucocorticoid duration. Additionally, creating separate arms for patients recovering from long-term glucocorticoid use can help isolate the impact of glucocorticoids on treatment response, minimizing confounding effects. Furthermore, statistical models, such as survival analysis or regression, can further control for glucocorticoid duration, ensuring more accurate evaluations of PD-1 therapy efficacy.

Ultimately, educating clinical trial stakeholders, including researchers, trial sponsors, and protocol designers, on these strategies will improve glucocorticoid management in trials, enhancing patient safety and ensuring more consistent, reliable PD-1 therapy outcomes.
